# Cytokeratin 18 as a Novel Biomarker in Patients with Hypertrophic Cardiomyopathy

**DOI:** 10.3390/cells13161328

**Published:** 2024-08-09

**Authors:** Konstantinos Fragkiadakis, Niki Ktena, Aikaterini Kalantidou, Eirini Dermitzaki, Ioannis Anastasiou, Stamatis Papathanassiou, Joanna Kontaraki, Petros Kalomoirakis, Emmanuel Kanoupakis, Alexandros Patrianakos, Antonis Papadomanolakis, Efsevia Daskalaki, Theodora Kiousi, Katerina Kouraki, Elena Kranioti, Maria Tzardi, Maria Venihaki, Domna Karagogeos, Yassemi Capetanaki, Dimitris Kardassis, Georgios Kochiadakis, Fragkiskos Parthenakis, Maria Marketou

**Affiliations:** 1 Cardiology Department, Heraklion University General Hospital, 71110 Heraklion, Greecekanoup@gmail.com (E.K.); maryemarke@yahoo.gr (M.M.); 2 Cardiology Department, School of Medicine, University of Crete, 71003 Heraklion, Greece; kontarai@uoc.gr; 3 Division of Basic Sciences, School of Medicine, University of Crete, 71003 Heraklion, Greecekaragoge@imbb.forth.gr (D.K.); 4 Clinical Chemistry, School of Medicine, University of Crete, 71003 Heraklion, Greece; katekld7@gmail.com (A.K.); venycham@uoc.gr (M.V.); 5 Institute of Molecular Biology, 55128 Mainz, Germany; 6 Forensic Medicine Unit, Department of Forensic Sciences, School of Medicine, University of Crete, 71003 Heraklion, Greece; 7 Laboratory of Pathology, University General Hospital of Heraklion, 71110 Heraklion, Greece; 8 Center of Basic Research, Biomedical Research Foundation, Academy of Athens, 11527 Athens, Greece; 9 Laboratory of Biochemistry, School of Medicine, University of Crete, 71003 Heraklion, Greece

**Keywords:** hypertrophic cardiomyopathy, cell death, cytokeratin-18, M30 antigen, M65 antigen, biomarkers

## Abstract

Hypertrophic cardiomyopathy (HCM) is a heart muscle disease associated with an increased risk for sudden cardiac death (SCD). Cytokeratin 18-based proteins, such as M30 and M65 antigens, are known cell-death biomarkers. M30 antigen is released from cells during apoptosis, and M65 antigen is released during cell death from any cause, such as apoptosis or necrosis. We aimed to study the expression of M30 and M65 antigens in peripheral blood obtained by 46 HCM patients and compare with 27 age- and sex-matched patients without HCM. We also investigated the CK18 expression in myocardium from postmortem HCM hearts. M30 and M65 antigens were significantly increased in the HCM vs. non-HCM group (Μ30: 338 ± 197 U/uL vs. 206 ± 166 U/uL, *p* = 0.003; M65: 428 ± 224 U/uL vs. 246 ± 214 U/uL, *p* = 0.001), and HCM patients with a higher expression of these markers (M30: 417 ± 208 vs. 271 ± 162 U/uL, *p* = 0.011; M65: 518 ± 242 vs. 351 ± 178 U/uL, *p* = 0.011) had a higher risk for SCD. In HCM, both apoptosis and necrosis are increased, but particularly necrosis (M30/M65 ratio: 0.75 ± 0.09 vs. 0.85 ± 0.02, *p* < 0.001). CK18 is expressed in the HCM myocardium (1.767 ± 0.412 vs. 0.537 ± 0.383, % of area, *p* = 0.0058). Therefore, M30 and M65 antigens may be novel biomarkers in HCM.

## 1. Introduction

Hypertrophic cardiomyopathy (HCM) is the most common genetic cardiomyopathy, with a prevalence of 1 in 200 [1,2]. It is characterized by unexplained left ventricular hypertrophy, impaired muscle fiber architecture, and myocardial fibrosis [3,4]. Mutations of the genes expressing sarcomeric proteins are the main cause of the disease and make the cardiomyocyte vulnerable to damage [5]. While most patients with HCM are asymptomatic, a subgroup of diseased individuals may present with sudden cardiac death (SCD) or severe heart failure. Therefore, biomarkers of myocardial damage or wall stress could be used as predictors for the occurrence of SCD or heart failure.

Mounting evidence suggests that cardiomyocytes’ cytoskeleton is crucial for normal myocardium function and heart disease development despite its small volume within the cell. The cytoskeleton is a complex filamentous and tubular network that maintains cell structure and transmits stimuli within and between cardiomyocytes. It is composed of a sarcomeric and non-sarcomeric cytoskeleton. The latter comprises cytoplasmic actin, microtubules, and intermediate filaments (IFs). The IF family comprises numerous proteins that exhibit tissue and differentiation-specific expression. Desmin is the muscle-specific IF, although other cytoskeletal IFs, like cytokeratins, may also be found under certain circumstances [6,7,8].

Cytokeratins 8 and 18 (CK8, CK18) are cytoplasmic keratin IFs expressed in simple epithelia [9]. However, growing evidence shows that they are also expressed in the myocardium during fetal heart development and heart diseases such as ischemic cardiomyopathy and heart failure [7,10,11]. Besides their mechanical role as a part of the cytoskeleton, they are involved in cell signaling, regulating the availability of other abundant cellular proteins, trafficking proteins, and acting like stress-proteins [12].

Numerous studies have demonstrated that cell death is an important component in the pathogenesis of myocardial infarction and heart failure. The evidence that apoptosis and necrosis play a role in heart disease renewed the interest in cell death and which anti-death therapy would be appropriate [13]. In chronic heart failure, an increased expression level of proinflammatory cytokines in peripheral circulation and within the myocardium is associated with the development of end-stage disease [14]. Tumor necrosis factor alpha (TNFa) is a proinflammatory cytokine that activates downstream cell-death signaling pathways that activate caspases and promote cardiomyocyte apoptosis [15]. During apoptosis, active effector caspases cleave intracellular substrates, including various cytoskeletal proteins [16,17,18,19], leading to aggregate formation, cytoplasmic budding, nuclear condensation, and the formation of apoptotic bodies. In a failing heart, desmin is cleaved by caspases activated by TNFa and forms cytoplasmic aggregates [17]. Similarly, during an epithelial cell apoptosis, caspase-9 dependent activation of caspase-3 and -7 induces the cleavage of the COOH-terminal fragment of CK18 and the formation of a new epitope, known as M30 antigen, which is recognized by a specific monoclonal antibody [18]. In contrast to apoptosis, necrosis is characterized by intracellular ATP depletion that impairs membrane function and provokes cellular and organelle swelling. It is usually accompanied by releasing intracellular components that induce inflammation and, finally, fibrosis [13]. It is widely known that proteins or their products are released in the bloodstream after cell death, particularly necrosis. M30 antigen has already been used as a selective apoptosis biomarker for pharmacodynamic cancer studies [20,21]. On the other hand, the M65 antigen is a marker of both apoptosis and necrosis since it recognizes both apoptotic M30 antigen and intact CK18, which are released after cell death [21]. Since there is evidence that CK18 is expressed in the myocardium of a failing heart by a TNFa-dependent pathway, the expression level of caspase-cleaved CK18 (M30 antigen) and intact CK18 in blood serum could estimate the cell death pattern in heart disease and provide diagnostic or prognostic tools to practitioners and guide therapy. However, limited studies have evaluated CK18 levels in patients with myocardial hypertrophy.

In the present study, we aimed to investigate M30 and M65 antigen levels in the serum obtained by HCM patients compared to non-HCM controls and investigate the type of cell death. We also intended to provide further evidence of the role of cell death by examining the association of M30 and M65 antigen levels with the disease severity and risk for heart failure or arrhythmias.

## 2. Materials and Methods

### 2.1. Study Population

We included patients with a confirmed diagnosis of HCM, aged between 18 and 65 years. The diagnosis was based on echocardiographic findings such as unexplained left ventricular hypertrophy with more than 15 mm wall thickness that was not caused by any contributing hemodynamic factor like aortic stenosis or any systemic infiltrative disease with heart involvement [22]. Individuals without HCM with similar age (within 5 years), sex, and cardiovascular risk factors were served as a control group (non-HCM group). An additional comparison was performed between the HCM group and healthy age and sex-matched participants without cardiovascular risk or disease (normal group). A comprehensive personal and family medical history was collected from all the participants. Our cohort was also composed of HCM patients with implantable cardioverter defibrillators (ICD) for primary prevention of sudden cardiac death (SCD). We excluded HCM patients with severe chronic kidney disease, diabetes mellitus with target-organ damage, pregnancy, or a history of septum reduction therapy, or an HCM-like phenotype due to infiltration or metabolic storage diseases such as amyloidosis and Fabry disease. Moreover, we excluded individuals with previous acute coronary syndrome, signs or history of coronary artery disease (CAD), severe valvular disease, history of cancer, liver and gall-bladder disease, severe chronic obstructive pulmonary disease, known autoimmune disease, and recent hospitalization for severe sepsis, major trauma, or surgery in the last month. A workout with stress cardiac imaging or coronary angiography was performed to exclude CAD as needed.

### 2.2. Echocardiography

A comprehensive echocardiography was performed on all the participants. A complete morphological and functional systolic assessment of the left ventricle (LV) was accomplished [23]. A standard M-mode and 2-dimensional (2D) echocardiography was performed using a Vivid E9 (General Electric Health Medical, Horten, Norway) ultrasound device with a 1.5 to 3.6 MHz wide-angle phased-array transducer (M4S), according to the recommendations of the American Society of Echocardiography and the European Association of Echocardiography [24], in order to measure the left ventricular end-diastolic (LVEDD) and end-systolic (LVESD) diameters, the thickness of the interventricular septum (IVS) and posterior wall (PW), the site and grade of maximal wall thickness (MWT), and the left ventricular ejection fraction (LVEF) [24]. 

Additionally, pulsed-wave (PW) Doppler was used to assess the mitral inflow pattern. Peak early diastolic mitral inflow velocity (E) and peak late diastolic mitral inflow velocity (A) were measured. Pulsed tissue Doppler imaging (TDI) from the apical 4-chamber view was used to record the longitudinal movement of the medial and lateral mitral annulus. The individual average from 6 measurements was used for each analysis. We measured annular septal and lateral early diastolic tissue velocities and calculated their average (e′). Then, the ratio of early diastolic mitral inflow velocity to early diastolic mitral annulus velocity (E/e′) was also determined for diastolic assessment. LV mass was calculated according to the Penn convention and expressed as LV mass index (LVMI) [22]. The left atrium (LA) anteroposterior diameter was measured from the parasternal long-axis view and LA volume from apical 4- and 2-chamber views, at left ventricular end-systole, using the Simpson’s biplane method, indexed to body surface area. Furthermore, the presence and the maximal gradient of LV outflow tract obstruction were determined at rest and after the Valsalva maneuver. 

Speckle-tracking echocardiography was used to calculate the average peak LV global longitudinal strain (GLS) from the three standard apical views (apical 3-, 2-, and 4-chamber views) [24]. GLS describes the relative change in the length of a myocardial speckle between end-systole and end-diastole and, therefore, is a negative number. Furthermore, we measured the LA strain, which describes the function for all phases of LA function, such as the reservoir, the conduit, and the atrial contraction [25]. The onset of QRS was used as the zero-reference point for LA strain calculation. 

### 2.3. Laboratory Examination

Blood samples were collected from all the participants on the same day as the echocardiography study after a 12 h fasting period. A total blood count and a complete biochemical examination were performed. Moreover, high-sensitive c-reactive protein (hs-CRP) and specific cardiac biomarkers such as high-sensitive troponin I (hs-TnI) and brain natriuretic peptide (BNP) were measured (Beckman Coulter Diagnostics, Brea, CA, USA). Samples of blood serum from each participant were also stored at −20 °C, and the expression of CK18, caspase-cleaved CK18, and TNFa were measured from each sample in duplicates using enzyme-linked immunosorbent assays (ELISA). Caspase-cleaved CK18, also known as M30 antigen, is a marker of apoptosis, and its expression was measured with the M30 Apoptosense^®^ ELISA kit (Prod. No. 10011, PEVIVA, Nacka, Sweden). The M65^®^ ELISA kit (Prod. No. 10020, PEVIVA, Nacka, Sweden) was used to measure the expression of total soluble extracellular CK18, which includes intact, full-length CK18 and its caspase-cleaved product (M30 antigen) and, therefore, is a marker of both apoptosis and necrosis. Finally, TNFa expression in blood serum was calculated using the Human TNFa ELISA kit (Cat. No. 430207, BIOLEGEND, San Diego, CA, USA). 

### 2.4. SCD Risk Assessment and Classification in HCM

In HCM, the risk assessment for SCD is based on several variables such as age, history of unexplained syncope, LV outflow tract gradient, maximum LV thickness, presence of NSVT, family history of SCD, LV systolic function, and myocardial fibrosis [26]. In our study, all the appropriate data were collected to calculate the 5-year risk for SCD for each HCM patient using the ESC risk calculator for SCD [27]. According to the 2023 ESC Guidelines, our HCM patients were classified in a high-risk group when the score was higher than or equal to 6% and in a non-high-risk group when it was below 6% [26]. 

### 2.5. Immunohistochemistry of Human Postmortem Heart Tissue 

We investigated the expression of CK18 in postmortem paraffin-embedded myocardial specimens obtained from the archives of the Forensic Medicine Unit of the University of Crete. Left ventricular tissue specimens from patients with HCM who died from sudden cardiac death were compared with three normal hearts from individuals who suffered death from a head injury. Criteria for HCM diagnosis in the heart specimens were asymmetric hypertrophy of the interventricular septum, with a maximal wall thickness of ≥15 mm and no history of hypertension, along with typical structural histochemical changes found in HCM myocardium such as cardiomyocyte hypertrophy with disarray, and interstitial fibrosis [28]. The CK18 expression was investigated at the interventricular septum of the left ventricle at both HCM and normal hearts. The clinical characteristics of the postmortem HCM and normal hearts are presented in Supplementary Table S1. 

Liver sections were used as a positive control for CK18 expression. The samples were embedded in paraffin, and a deparaffinization protocol was applied to enable antibody penetration. After dewaxing, heat antigen retrieval was performed with an EDTA-based solution. Following antigen retrieval, the sections were incubated with a blocking solution (5% BSA, 0.5% Triton-X in 0.1 M PBS) for 1 h at room temperature for immunofluorescence. Subsequently, the primary antibodies for human cytokeratin 18 (1:200, mouse, ab7797, Abcam, Cambridge, UK) and desmoplakin (1:200, rabbit, ab71690, Abcam, Cambridge, UK) diluted in the same blocking solution were applied, and the sections were incubated overnight at 4 °C in a humidified chamber. The next day, the sections were incubated with secondary antibodies Alexa Fluor 555 (1:800, anti-mouse, Cat# A-21422, ThermoFisher, Waltham, MA, USA) and CFTM 488A (1:800, anti-rabbit, Cat# 20012, Biotium, Fremont, CA, USA) for 2 h at room temperature and counterstained with DAPI (Cat# D1306, ThermoFisher, Waltham, MA, USA) to visualize the nuclei. Finally, all the slides were mounted using a Mowiol mounting medium (Merk-Millipore, Burlington, MA, USA), and image acquisition was performed in a confocal microscope (TCS SP8, LEICA DMI-8, Leica, Wetzlar, Germany).

#### Analysis of Confocal Images

Immunohistochemistry was performed on the same day for all the sections for cell fluorescence and intensity measurements. All the images were captured using the same image acquisition settings: ×40 magnification, 1 μm step size, high resolution (1024 × 1024 pixels), identical laser intensity, and an identical number of z-stacks for each image, using a TCS SP8 laser scanning confocal microscope. Following image acquisition, the images were analyzed by Fiji ImageJ software (https://imagej.net/software/fiji/downloads) and quantified blindly to the result. 

### 2.6. Statistical Analysis

Continuous variables were presented as mean ± standard deviation (mean ± SD), while categorical variables were presented as frequencies and group percentages. For comparing two independent groups, the Student’s *t*-test was used for parametric data, and the Mann–Whitney U test for nonparametric data. For categorical variables, a Fisher’s Chi-square was performed. Furthermore, ROC calculations were performed where appropriate. All the statistics were performed using SPSS software (IBM SPSS Statistics, version 26). Image J software (https://imagej.net/software/fiji/downloads) was used to estimate the CK18 immunostaining quantitatively in the postmortem myocardium. Finally, all *p*-values were 2-sided; a statistically significant difference was considered when the *p*-value was <0.05. 

### 2.7. Ethical Approval

The study protocol was approved by the Ethics Committee of the University of Crete, Greece, and it was in concordance with the ethical principles stated in the Declaration of Helsinki. Furthermore, all the participants gave informed consent, and the recommended institutional guidelines were followed. The postmortem samples were acquired retrospectively from the archives of the Forensic Medicine Unit of the University of Crete, and were collected for routine histopathology examinations during autopsies. No informed consent was necessary for this purpose. 

## 3. Results

### 3.1. Characteristics of HCM and Non-HCM Group

**Figure 1:**
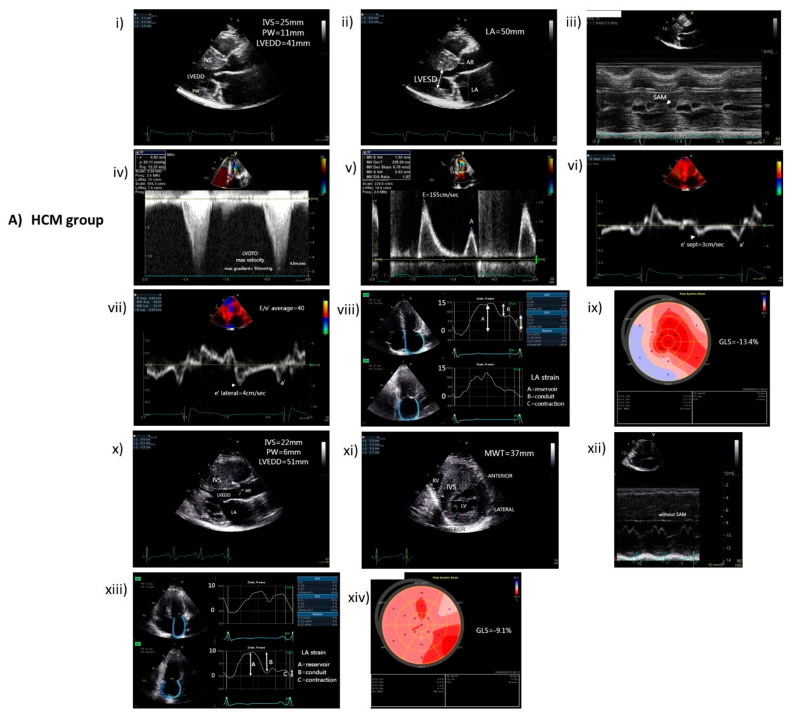

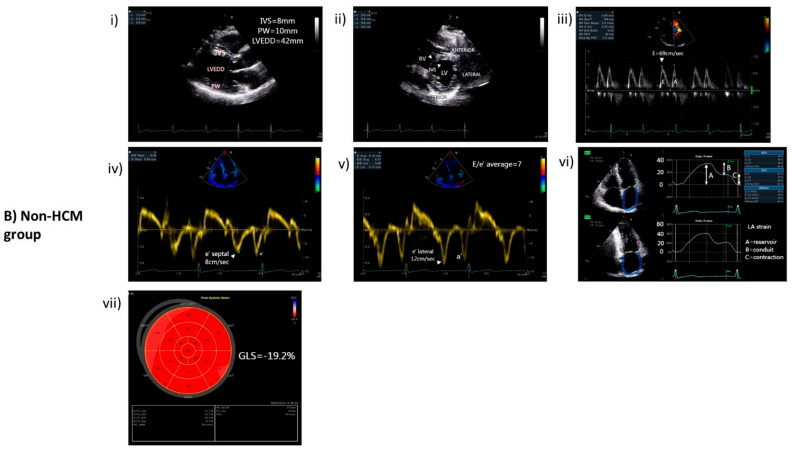
Representative echocardiographic images from HCM and non-HCM groups. (**A**) (i–ix) images from a patient with obstructive HCM; (i) 2D PLAX view at end-diastole, (ii) 2D PLAX view at end-systole, (iii) M-Mode through the LVOT, (iv) CW Doppler through LVOT, (v) PW Doppler of mitral inflow, (vi–vii) Doppler tissue imaging velocities of the medial and lateral mitral annulus, respectively; (viii) LA strain, (ix) LV GLS; Note the asymmetric hypertrophy of anterior IVS, the dilated anteroposterior diameter of LA, the SAM of the mitral valve that causes LVOT obstruction with a maximum PG of 93 mmHg at rest, and the diastolic dysfunction with a restrictive mitral inflow pattern and low diastolic tissue velocities. Moreover, observe the low absolute values of the LA strain and LV GLS. (**A**) (x–xiv) images from a patient with non-obstructive HCM; (x) 2D PLAX view at end-diastole, (xi) 2D SAX view at the level of papillary muscles at end-diastole, (xii) M-Mode through LVOT, (xiii) LA strain, (xiv) LV GLS, Note the marked thickening of the anterior IVS, the absence of SAM of the mitral valve and LVOT obstruction at rest or after a Valsalva maneuver. Furthermore, observe the low absolute values of the LA strain and LV GLS. (**B**) (i–vii) images from a patient without HCM; (i) 2D PLAX view at end-diastole, (ii) 2D SAX view at the level of papillary muscles upon end-diastole, (iii) PW Doppler of mitral inflow, (iv-v) Doppler tissue imaging velocities of the medial and lateral mitral annulus, respectively; (vi) LA strain, (vii) LV GLS. Note the normal shape and thickness of LV, the normal mitral inflow pattern and LV tissue velocities. Observe the normal absolute values of LA strain and LV GLS. **Abbreviations**: HCM: hypertrophic cardiomyopathy; 2D: two-dimensional ultrasound; PLAX: parasternal long-axis view; LVOT: left ventricular outflow tract; CW: continuous-wave Doppler; PW: pulsed-wave Doppler; PG: peak gradient; LA: left atrium; LV: left ventricle; SAM: systolic anterior motion of mitral valve; GLS: global longitudinal strain; IVS: interventricular septum; SAX: short-axis view.

This study included 46 HCM patients and 27 age- and sex-matched controls. The mean age of the HCM group was 50 ± 11 years old; 36 were males, and 10 were females. Among the two groups (HCM vs. non-HCM), there was not any significant difference between the body mass index (29.8 kg/m^2^ vs. 29.5 kg/m^2^, *p* = 0.807); traditional cardiovascular risk factors, such as smoking (45.7% vs. 44.4%, *p* = 1.000), diabetes mellitus (19.6% vs. 14.8%, *p* = 0.756), hypertension (45.7% vs. 40.7%, *p* = 0.808), dyslipidemia (54.3% vs. 63%, *p* = 0.624); or presence of atrial fibrillation (23.9% vs. 22.2%, *p* = 1.000). Baseline demographic, clinical, and laboratory data are presented in Table 1. Furthermore, the corresponding echocardiographic characteristics of HCM and non-HCM groups are also presented in Table 2. Representative echocardiographic images from each group (HCM vs. non-HCM) are found in Figure 1. 

### 3.2. Levels of CK18 and Its Caspase-Cleaved Product in the Blood Serum of Patients with HCM

**Figure 2:**
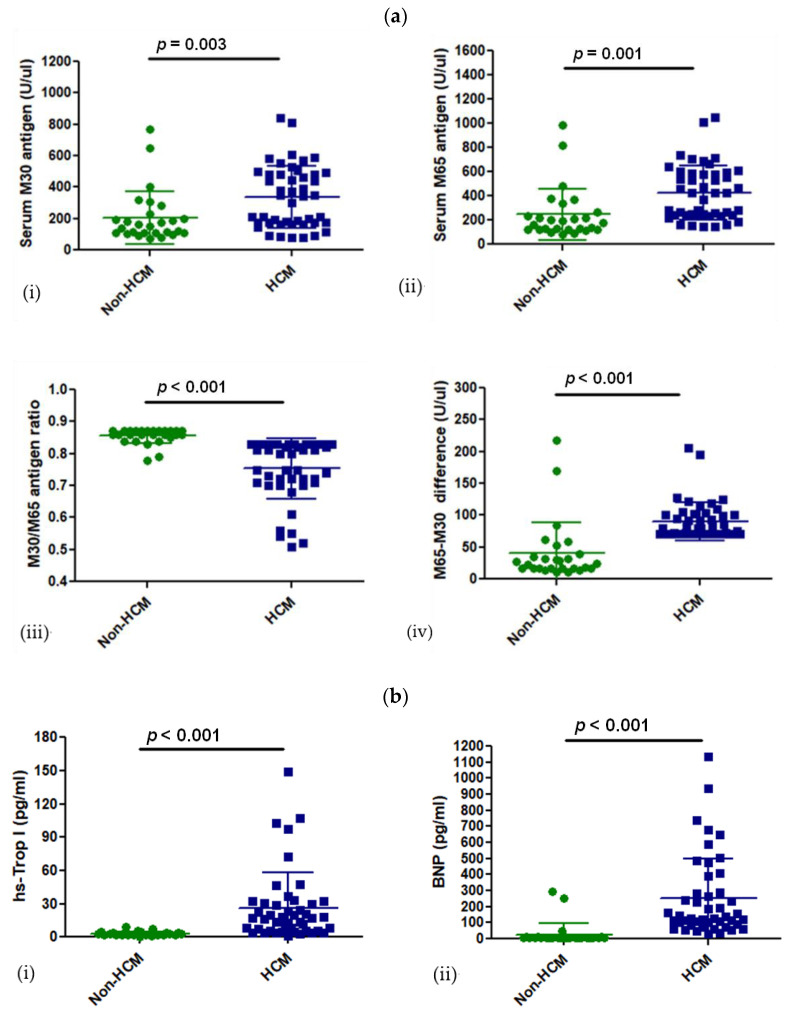
Expression of CK18-based biomarkers and other cardiac biomarkers in HCM vs. non-HCM control group. (**a**) Expression of M30 (i) and M65 (ii) antigens along with their ratio (M30/M65 antigens ratio) (iii) and their difference (M65-M30) (iv), in the blood serum of patients with HCM and non-HCM (control-group), (**b**) hs-TnI (i) and BNP (ii) values are also presented among the two groups.

CK18 and its caspase-cleaved product, M30 antigen, were measured in the blood serum of patients with HCM or their age- and sex-match controls. M30 antigen—a marker of apoptosis—was significantly increased in patients with HCM compared to the control group (338 ± 197 U/uL vs. 206 ± 166 U/uL, *p* = 0.003). The M65 antigen—a marker of total cell death (apoptosis and necrosis)—was significantly higher in HCM than in the control group (428 ± 224 U/uL vs. 246 ± 214 U/uL, *p* = 0.001) (Figure 2a). The difference between M65 and M30—a necrosis marker—was significantly higher in patients with HCM than in the control group (90 ± 29 U/uL vs. 40 ± 47 U/uL, *p* < 0.001) (Figure 2a). Therefore, there seems to be more necrosis in the HCM group than in the control group, which is also supported by the significantly lower M30 to M65 ratio (0.75 ± 0.09 vs. 0.85 ± 0.02, *p* < 0.001) (Figure 2a). Since there is knowledge that cytokeratin 18 expression depends on TNFa expression, the level of TNFa expression was investigated in the peripheral blood of HCM patients and the control group. However, the TNFa expression level was undetectable in serum samples of both HCM and control groups (Human TNFa ELISA kit, sensitivity 3.5 pg/mL). Overall, the increase in M30 and M65 antigen expression levels was significant in HCM, and it follows the expected significant difference of cardiac-specific markers like hs-trop I (HCM vs. control; 26 ± 31 pg/mL vs. 3 ± 2 pg/mL, *p* < 0.001) and BNP (250 ± 252 pg/mL vs. 28 ± 72 pg/mL) among the 2 groups (Figure 2b). Although serum hs-CRP was increased in the HCM group compared with the non-HCM group (0.53 ± 0.69 mg/dL vs. 0.25 ± 0.28 mg/dL), this increase was almost not statistically significant (*p* = 0.05) (Table 1). 

Moreover, we compared the expression of each M30 and M65 antigen and their difference and ratio in patients with HCM and 10 normal individuals. The results showed significant differences between the two groups (HCM vs. normal; M30 antigen: 337 ± 197 U/uL vs. 177 ± 103 U/uL, M65 antigen: 427 ± 224 U/uL vs. 208 ± 126 U/uL, M65-M30: 90 ± 29 U/uL vs. 31 ± 23 U/uL, M30/m65 ratio: 0.75 ± 0.09 vs. 0.86 ± 0.01, *p* <= 0.001 for all) and are presented in the supplementary data (Supplementary Figure S1).

### 3.3. M30 and M65 Expression in HCM Group with or without High Risk for SCD

**Figure 3:**
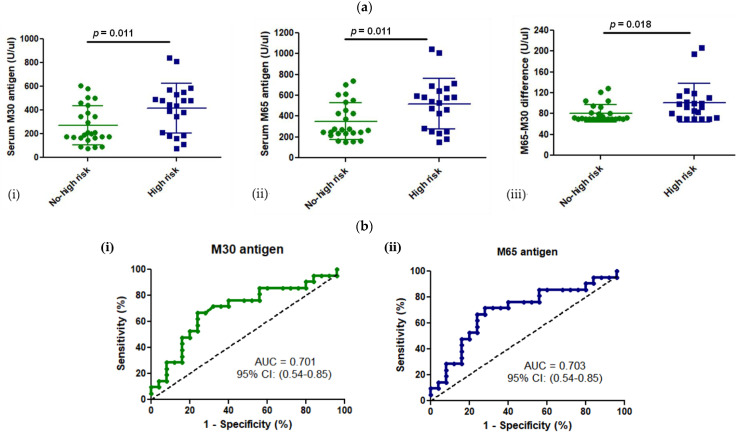
(**a**) Expression of CK18-based biomarkers in HCM patients with or without high risk for SCD. (i) M30 antigen, (ii) M65 antigen, (iii) M65-M30 difference (**b**) M30 (i) and M65 (ii) antigens’ ROC analysis to identify high-risk HCM patients for SCD.

According to the ESC HCM Risk-SCD score, 21 patients (46%) had a high risk for SCD within the HCM group (n = 46 patients) and 25 patients (54%) did not. Among our HCM patients, 21 had an ICD, while 25 did not. The expression of M30 and M65 antigens was significantly higher in patients with a high risk for SCD than HCM patients without, respectively (M30: 417 ± 208 U/uL vs. 271 ± 162 U/uL, *p* = 0.011; M65: 518 ± 242 U/uL vs. 351 ± 178 U/uL, *p* = 0.011) (Figure 3a). Additionally, patients with a high risk for SCD had a higher level of the necrotic marker, intact-CK18, expressed by the difference between M65 and M30 (M65-M30: 101 ± 37 U/uL vs. 81 ± 17 U/uL, *p* = 0.018). Therefore, HCM patients with a high risk for SCD had a higher expression of cell death markers in the peripheral blood. 

Furthermore, a receiver operating curve (ROC) analysis was performed to investigate whether M30 or M65 antigen expression could be used as a diagnostic test to identify those HCM patients with a low or high risk for life-threatening ventricular arrhythmias and SCD. According to the ROC curves, both tests seemed to have a moderate but statistically significant power (Area Under the Curve or AUC of 0.70 for M30 or M65 antigen) to discriminate HCM patients with an increased risk for SCD (Figure 3b).

### 3.4. Tissue Expression of CK18 in Postmortem Myocardial Specimens from an HCM or a Normal Heart

**Figure 4:**
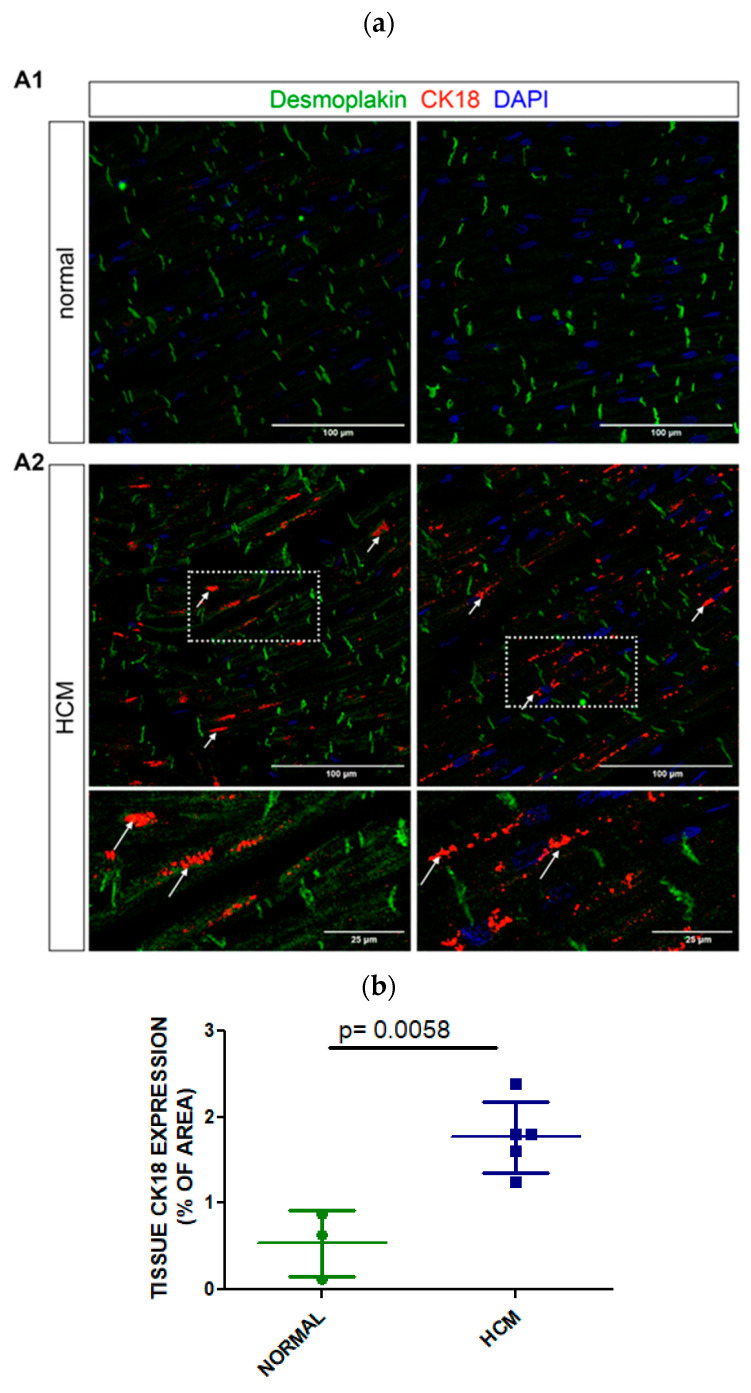
CK18 expression in human myocardium (**a**) Representative confocal images of human control (non-HCM, A1) and HCM (A2) myocardium, immunolabeled for CK18 (red) and desmoplakin (green). Nuclei are stained with DAPI (blue). White arrows indicate the CK18 aggregates. Scale bar: 100 µm. (**b**) Quantitative analysis of CK18 immunostaining from 5 HCM and 3 normal hearts.

The expression of CK18 was investigated in the postmortem myocardium of 5 individuals with hypertrophic cardiomyopathy and 3 individuals with normal hearts (Figure 4a). CK18 was significantly expressed in HCM cardiomyocytes, along the myofiber, and potentially at the costameres of the sarcolemma in postmortem myocardium tissue. In the HCM myocardium, CK18 created local formations that looked like aggregates in sarcolemma (Figure 4a, shown with white arrows). Co-staining with desmoplakin did not demonstrate co-localization at the intercalated disks in the postmortem myocardium. Quantitative analysis of confocal imaging data for CK18 immunostaining demonstrated that CK18 was significantly expressed in HCM myocardium compared to normal (1.767 ± 0.412 vs. 0.537 ± 0.383, % of area, *p* = 0.0058) (Figure 4b). Moreover, HCM patients whose cause of death was cardiac had a nonsignificant trend to express more CK18 at the tissue level than those with noncardiac death (Supplementary Data, Table S1). Therefore, the expression of CK18 in HCM myocardium might be the source of increased expression levels of CK18-based biomarkers (M30, M65 antigen) in the peripheral blood of HCM patients.

## 4. Discussion

Our study is the first to investigate the expression of CK18-based cell death markers in HCM and their association with the clinical characteristics of the disease. We found that M30 and M65 cell death markers are significantly increased in the blood serum of patients with HCM compared to those without. Although there is a significant increase in apoptosis and necrosis CK18-based serum biomarkers in HCM, the amount of necrosis is relatively higher in the HCM group compared to the non-HCM group. Furthermore, HCM patients with an increased expression of M30 and M65 antigens have a higher risk for SCD. 

CK18 is a member of the epithelial keratin type I, acidic group, intermediate filament protein. Along with a type II keratin group member, usually CK8, they form heterodimers that polymerize and form the keratin filaments [29]. In contrast with the “hard” extracellular keratins that compose hair and nails, CK18 and CK8 are “soft” cytoplasmic keratins and comprise a cytoplasmic filamentous network that connects the nucleus, the organelles with specific adhesive structures found on the epithelial cell cytoplasmic membrane. These structures include desmosomes and hemidesmosomes that participate in cell-to-cell and cell-to-matric interaction. In addition to their mechanical role, cytokeratins are involved in cell signaling, organelle function, and stress response [9]. CK8 and 18 proteins are expressed principally in single-layered epithelia found in the liver, kidney, pancreas, and intestine. However, their expression is not limited to simple epithelia, and there is growing evidence that they are expressed in other cells to compensate for a cellular injury. Recently, Papathanasiou et al. identified that CK8 and CK18 are ectopically expressed in cardiomyocytes upon NFKb-mediated stress stimuli to maintain intercalated disk (ID) integrity and mitochondrial function, playing a significant cardioprotective role [7]. 

The expression of CK18 in fetal myocardium was first described by the end of the 1980s [10]. The myocardium is derived from a polarized epithelium comprising cytokeratin IFs and desmosomes. CK18 has a cytoplasmic and membranous localization, mostly around desmosomes, with a punctuated pattern. During cardiac myogenesis, the muscle-specific IF desmin is expressed abundantly, and it is finally present in adult myocardium, while CK18 has disappeared [30]. 

In a normal adult cardiomyocyte, desmin is the principal IF protein. It creates a 3D scaffold, linking myofibers along the sarcomere’s Z-lines with mitochondria, nucleus, and desmosomes at intercalated disks on the intercellular junction [6,8]. Desmoplakin is the key protein that connects desmin with desmosomes. The desmin’s IF network also interacts with costameres, specialized plasma membrane complexes above the Z-lines, and connects intracellular apparatus (organelles and myofibers) with the extracellular matrix. In addition to its mechanical role, desmin’s IF network senses extracellular mechanical stress and transduces intracellular signals to activate pathways that maintain cardiomyocyte homeostasis [6,8]. The essential role of IF cytoskeleton in cardiomyocytes is recognized in desmin deficiency or disruption. In a heart-failure mouse model, desmin was cleaved by caspase-6, losing its mechanical and nonmechanical properties and forming cytoplasmic aggregates [17]. In general, an injury of the native IF cytoskeleton may activate the overexpression of native IF genes or other IF members to compensate for the mechanical stress. Indeed, overexpression of the CK8 and CK18 IF networks in a mouse model with desmin deficiency restored ID disruption, myofiber disarray, and mitochondrial dysfunction [7]. 

In the last two decades, oncology has increasingly utilized CK18-based cell death markers for disease prognosis or therapeutic response. The availability of convenient assays to determine their expression level in serum has expanded their utilization. However, their utilization in cardiology is limited because not enough is known about the role of CK18 in heart disease. Despite the knowledge that CK18 is almost absent in adult human cardiomyocytes under normal conditions, there is growing evidence that it is found in heart disease. CK18 and ccCK18 (or M30 antigen) are expressed within the cardiomyocytes of patients with ischemic, hypertrophic, and congestive heart failure [7,11]. Particularly, ccK18 is found within cardiomyocyte lysosomes of ischemic myocardium and is a significant marker of myocardial damage and severity of coronary artery disease [31,32]. On the other hand, increased IgG antibodies against CK18 have been associated with an increased risk of coronary artery disease and cardiovascular events in patients with rheumatoid arthritis [33]. In addition to ischemic heart disease, CK18 and ccCK18 levels are severely elevated after an episode of acute heart failure compared to chronic heart failure, and they are associated with liver congestion and injury. It seems that necrosis plays a more significant role in acute heart failure and liver injury than apoptosis [34]. Recently, there has been evidence that ccCK18 expression is associated proportionally with the degree of obesity and severity of diastolic dysfunction in adolescents [35]. Therefore, M30 and M65 antigens seem to be novel markers of cell death in heart disease, but not solely in cancer, since there is evidence that CK18 participates in the pathophysiological pathway of myocardial injury.

Numerous biomarkers have been studied in HCM over the last three decades to elucidate the pathophysiology of the disease and recognize those with an increased risk of end-stage heart failure or arrhythmias. According to the biological pathway in which they are implicated, they might be classified as inflammatory, endothelial, fibrosis, wall stress, or apoptosis biomarkers [36]. Evidence shows that increased blood expression of FAS family-apoptosis mediators or troponin T is associated with the dilated phase of HCM [37,38].

However, the role of cell death in the evolution of HCM is still under investigation. Moreover, it has not been investigated whether the type of cell death, apoptosis, or necrosis plays a role in the severity of the disease. Therefore, we aimed to investigate the type of cell death in HCM using the CK18-based cell death markers -M30 and M65 antigens- and their association with the disease. The strength of our study was the meticulous exclusion of any condition associated with elevated levels of CK18 or M30 antigen in the blood [33,39,40,41,42,43,44,45] and the recruitment of patients with mostly sarcomeric hypertrophic cardiomyopathy. We observed that the expression of M30 and M65 antigens was significantly increased in the serum of patients with HCM compared to those without HCM. The higher expression of these cell-death biomarkers might be associated with the higher LV hypertrophy and mass found in HCM that increase mechanical stretch and oxygen mismatch in hypertrophic myocardium, thus promoting cell death in HCM. Furthermore, we found a relative increase in necrosis in HCM compared to the control group, and this event might contribute to interstitial fibrosis in HCM myocardium since necrosis is known to promote inflammation and profibrotic pathways [13]. In our HCM group, there was a trend for higher expression of the inflammatory marker hsCRP than the control group, but it was insignificant. Moreover, it is noteworthy that the higher expression of CK18-based cell death biomarkers found in HCM followed the increased expression of our traditional biomarkers of myocardial wall stress and necrosis. Although TNFa is an inflammatory cytokine implicated in CK18 ectopic expression at cardiomyocytes, its expression was significantly low and not different both in the peripheral blood of HCM and non-HCM groups. This might be explained by the fact that TNFα was solely overexpressed at a myocardial level or had a rapid and transient release in peripheral blood, making it unmeasurable. Indeed, there is evidence that TNFa is not significantly increased in the peripheral blood circulation of hypertrophic cardiomyopathy [37,46]. However, TNFa is overexpressed when HCM progresses to the dilative type of HCM or end-stage burnout HCM [37,46]. Finally, the increased expression of CK18-based cell death biomarkers in HCM compared to the control group might also indicate a role of cell death in diastolic dysfunction. Overall, the higher amount of cellular death and particularly necrosis found in HCM may be implicated in the activation of inflammatory and fibrotic pathways during the disease’s natural history and associated with its severity. Indeed, in our study, we found that CK18-based cell death markers were elevated in patients with a high risk for SCD. An ROC analysis revealed that these biomarkers bear the power to identify those HCM patients with a higher risk for SCD.

Our study also confirmed that CK18 is significantly expressed in the myocardial tissue of HCM patients who suffered a cardiac or noncardiac death compared with normal individuals. CK18 did not form filaments but a punctuated pattern within the cardiomyocytes as previously described in fetal myocardium [10] and localized across the cytoplasm and near the costameres. However, it was not found near the IDs as previously presented by Papathanasiou et al. in the myocardium of patients with failing hearts who were candidates for heart transplantation [7]. The absence of filament formation and CK18 localization on IDs could be explained by the inadequate expression of type II CK8, which is obligatory for cytokeratin polymerization or the CK18 cleavage by caspases, with subsequent removal from IDs as shown in a postmortem myocardium. Furthermore, we observed a formation of local aggregates in HCM myocardium that could result from caspase activation during apoptosis. Moreover, we have observed that CK18 expression was higher in the myocardium of HCM patients with HCM who suffered from SCD compared to those who died from a head injury. Notably, the ectopic expression of CK18 in the myocardium seems to be part of the fetal genome program that is reactivated during heart disease [47].

M30 and M65 antigens are CK18-based biomarkers that could give insight into the possible type and grade of cell death in HCM. We showed that those HCM patients with an increased expression of serum CK18-based biomarkers had a high risk for SCD. It is still under investigation whether a more pronounced necrotic relative ratio or a higher expression of these biomarkers is associated with a more severe phenotype or progressive disease. Moreover, it should be investigated whether their expression could be used as targets in the therapeutic management of HCM. Therefore, more studies are needed to evaluate their potential role as prognostic biomarkers or therapeutic targets in HCM.

## 5. Limitations

We performed a single-center, cross-sectional study with a small number of HCM patients. Although our groups were small, our findings were clear. The serum CK18-based cell death biomarkers source could not be precisely identified because we examined their expression in peripheral blood rather than coronary sinus blood. Thus, the source of these serum biomarkers in HCM could be considered questionable. For this reason, we attempted to reduce uncertainty and improve the reliability of our results by excluding individuals with a noncardiac cause of CK18-based biomarker expression. Additionally, we compared our HCM group with a similar control group to ensure that the only distinction was the presence of HCM. Furthermore, the expression of CK18 within the cardiomyocytes of HCM patients increased the validity of our findings. Although the quality of postmortem tissue was limited by the postmortem interval and sample preservation, the expression of CK18 in the myocardium was clear. CK8 is the usual counterpart of CK18 in forming cytoskeletal filaments, but we didn’t investigate its expression in HCM myocardium because our study focused on CK18-based biomarkers. Although we did not conduct a complete genetic analysis of our HCM group for sarcomere mutations, excluding other genetic or nongenetic disorders that mimic HCM supports that our HCM group included mostly sarcomeric HCM. Moreover, the absence of a sufficient number of cardiac magnetic resonance data for myocardial fibrosis in our HCM group didn’t allow us to investigate its association with the expression level of CK18-based biomarkers. Finally, prospective observational studies with more participants are necessary to assess their prognostic role in the natural course of HCM.

## 6. Conclusions

**Figure 5:**
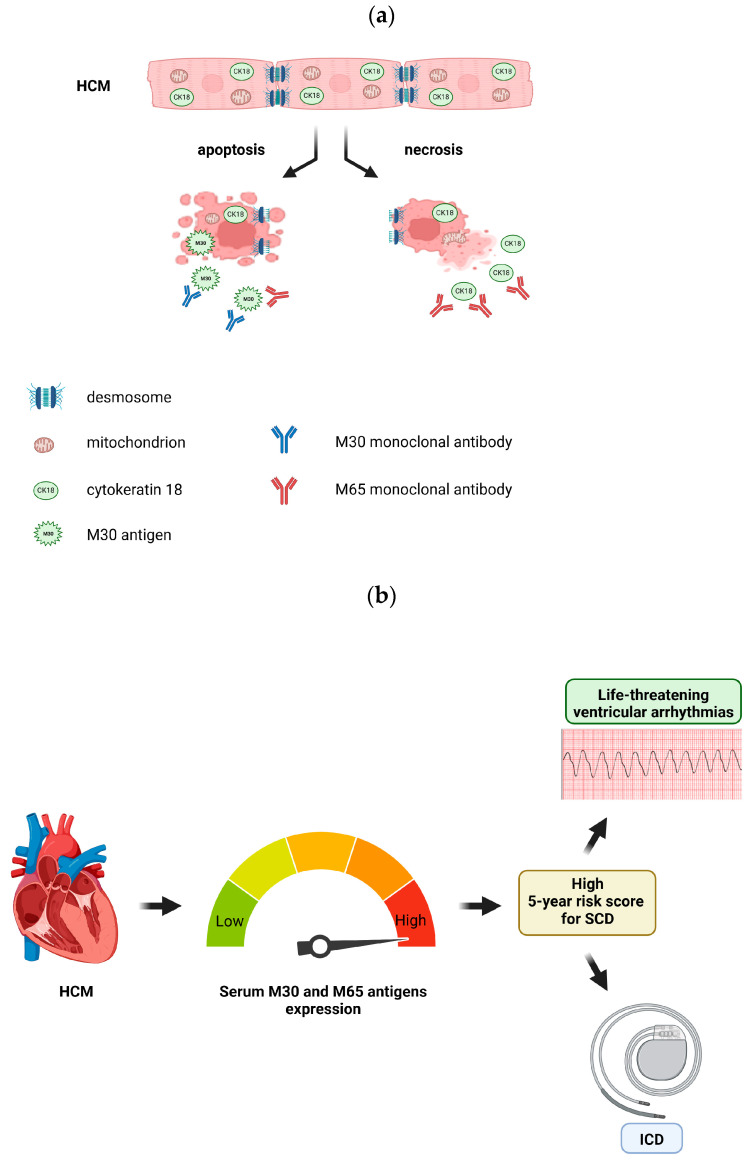
(**a**) The expression of the different types of CK18-based biomarkers measured with available immunoassays determines the type of cardiomyocyte death in HCM. (**b**) The level of M30 and M65 antigen expression is associated with the risk for life-threatening arrhythmias and sudden cardiac death in HCM. The images were created with BioRender.com.

Cytokeratin 18 (CK18), a member of the intermediate filament cytoskeleton, was ectopically expressed in cardiomyocytes of patients with HCM as a response to increased mechanical stress. In HCM, there was a significant release in the peripheral blood of CK18-based proteins, such as M30 and M65 antigens, which are markers of apoptosis and necrosis (Figure 5a). This significant cell-death increase in HCM, particularly necrosis, might be associated with LV hypertrophy, diastolic dysfunction, and disease progression. Furthermore, we identified that patients with higher levels of apoptosis and necrosis were at higher risk for SCD (Figure 5b). Further investigation is necessary with larger samples and prospective studies to evaluate whether these CK18-based cell death biomarkers could be prognostic tools in the management of hypertrophic cardiomyopathy.

## Figures and Tables

**Table 1:** Clinical characteristics and laboratory tests in HCM vs. non-HCM group.

	HCM (n = 46)	Non-HCM (n = 27)	Significance (*p*-Value)
Age (years)	50 (±11)	50 (±10)	0.731
Male/Female	36 (78.3%)/10 (21.7%)	20 (74.1%)/7 (25.9%)	0.777
BMI (kg/m^2^)	29.8 (±5)	29.5 (±6)	0.807
Smoking	21 (45.7%)	12 (44.4%)	1.000
Diabetes Mellitus	9 (19.6%)	4 (14.8%)	0.756
Hypertension	21 (45.7%)	11 (40.7%)	0.808
Dyslipidemia	25 (54.3%)	17 (63%)	0.624
Atrial Fibrillation	11 (23.9%)	6 (22.2%)	1.000
WBC (K/uL)	7.2 (±1.7)	7.9 (±2.5)	0.185
Hgb (mg/dL)	14 (±1)	14 (±1)	0.184
PLTs (K/uL)	225 (±60)	253 (±55)	0.054
SGOT (U/L)	28 (±11)	25 (±8)	0.157
SGPT (U/L)	33 (±22)	31 (±18)	0.748
gGT (U/L)	37 (±30)	29 (±14)	0.252
ALP (U/L)	73 (±22)	72 (±18)	0.754
TB (mg/dL)	0.8 (±0.7)	0.7 (±0.3)	0.460
Creatinine (mg/dL)	1.03 (±0.18)	1.01 (±0.20)	0.703
Hs-CRP (mg/dL)	0.53 (±0.69)	0.25 (±0.28)	0.050

Values are mean ± SD or n (%). **Abbreviations**: HCM: hypertrophic cardiomyopathy; BMI: body mass index; WBC: white blood cells; Hgb: hemoglobin; PLTs: platelets; SGOT: Serum glutamic-oxaloacetic transaminase; SGPT: Serum glutamic pyruvic transaminase; gGT: Gamma-glutamyl transferase; ALP: Alkaline phosphatase; TB: Total bilirubin; Hs-CRP: high-sensitive C-reactive protein.

**Table 2:** Echocardiographic parameters in HCM vs. non-HCM group.

	HCM (n = 46)	Non-HCM (n = 27)	Significance (*p*-Value)
LVEDD (mm)	47 (±7)	47 (±4)	0.893
LVESD (mm)	30 (±6)	31 (±4)	0.684
IVS (mm)	18 (±4)	10 (±2)	<0.001
PW (mm)	11 (±2)	9 (±1)	0.005
MWT (mm)	19 (±3)	10 (±2)	<0.001
LV mass index (g/m^2^)	137 (±30)	77 (±23)	<0.001
Location of asymmetric LV hypertrophy			
*IVS*	*39 (84%)*	-	
*Apex*	*4 (9%)*	-	
*Mixed*	*3 (7%)*	-	
LVOTO (mmHg)	14 (30%) 61 mmHg ± 35	-	
LVEF (%)	58 (±8)	60 (±4)	0.435
GLS (%)	−14 (±6)	−20 (±2)	<0.001
LA diameter (mm)	47 (±7)	39 (±5)	<0.001
LA volume index (mL/m^2^)	51(±18)	29 (±10)	<0.001
E/e’ratio	13 (±7)	8 (±3)	<0.001
LA strain (reservoir)	20 (±7)	31 (±9)	<0.001
LA strain (conduit)	−10 (±5)	−15 (±5)	0.001
LA strain (contraction)	−10 (±5)	−16 (±6)	<0.001

Values are mean ± SD or n (%). **Abbreviations**: HCM: hypertrophic cardiomyopathy; LVEDD: Left ventricular end-diastolic diameter; LVESD: Left ventricular end-systolic diameter; IVS: intraventricular septum; PW: posterior wall; MWT: maximal wall thickness; LV: left ventricle; LVOTO: Left ventricular outflow tract obstruction; LVEF: Left ventricular ejection fraction; GLS: global longitudinal strain: LA: left atrium; E/e’ ratio: ratio of early diastolic mitral inflow velocity to early diastolic mitral annulus velocity.

## Data Availability

Data are available after a reasonable request to the corresponding author.

## Supplementary Materials

The following supporting information can be downloaded at: https://www.mdpi.com/article/10.3390/cells13161328/s1. Figure S1: A comparison of the expression of the CK18-based biomarkers in the HCM group vs. a normal group with 10 normal individuals; Table S1: Clinical characteristics of the postmortem HCM or normal hearts and their associated quantitative data of CK18 immunostaining. Figure S2: The expression of CK18 in hepatocytes.

## References

[1] Maron B.J. (2010). Contemporary Insights and Strategies for Risk Stratification and Prevention of Sudden Death in Hypertrophic Cardiomyopathy. Circulation.

[2] Semsarian C., Ingles J., Maron M.S., Maron B.J. (2015). New Perspectives on the Prevalence of Hypertrophic Cardiomyopathy. J. Am. Coll. Cardiol..

[3] Maron B.J. (2002). Hypertrophic Cardiomyopathy: A Systematic Review. JAMA.

[4] Watkins H., Ashrafian H., Redwood C. (2011). Inherited Cardiomyopathies. N. Engl. J. Med..

[5] Olivotto I., Girolami F., Sciagrà R., Ackerman M.J., Sotgia B., Bos J.M., Nistri S., Sgalambro A., Grifoni C., Torricelli F. (2011). Microvascular Function Is Selectively Impaired in Patients with Hypertrophic Cardiomyopathy and Sarcomere Myofilament Gene Mutations. J. Am. Coll. Cardiol..

[6] Capetanaki Y., Papathanasiou S., Diokmetzidou A., Vatsellas G., Tsikitis M. (2015). Desmin Related Disease: A Matter of Cell Survival Failure. Curr. Opin. Cell Biol..

[7] Papathanasiou S., Rickelt S., Soriano M.E., Schips T.G., Maier H.J., Davos C.H., Varela A., Kaklamanis L., Mann D.L., Capetanaki Y. (2015). Tumor Necrosis Factor-α Confers Cardioprotection through Ectopic Expression of Keratins K8 and K18. Nat. Med..

[8] Tsikitis M., Galata Z., Mavroidis M., Psarras S., Capetanaki Y. (2018). Intermediate Filaments in Cardiomyopathy. Biophys. Rev..

[9] Coulombe P.A., Omary M.B. (2002). “Hard” and “Soft” Principles Defining the Structure, Function and Regulation of Keratin Intermediate Filaments. Curr. Opin. Cell Biol..

[10] Kuruc N., Franke W.W. (1988). Transient Coexpression of Desmin and Cytokeratins 8 and 18 in Developing Myocardial Cells of Some Vertebrate Species. Differentiation.

[11] Soleiman A., Lukschal A., Hacker S., Aumayr K., Hoetzenecker K., Lichtenauer M., Moser B., Untersmayr E., Horvat R., Ankersmit H.J. (2008). Myocardial Lipofuscin-Laden Lysosomes Contain the Apoptosis Marker Caspase-Cleaved Cytokeratin-18. Eur. J. Clin. Investig..

[12] Oshima R.G., Baribault H., Caulín C. (1996). Oncogenic Regulation and Function of Keratins 8 and 18. Cancer Metastasis Rev..

[13] Del Re D.P., Amgalan D., Linkermann A., Liu Q., Kitsis R.N. (2019). Fundamental Mechanisms of Regulated Cell Death and Implications for Heart Disease. Physiol. Rev..

[14] Hedayat M., Mahmoudi M.J., Rose N.R., Rezaei N. (2010). Proinflammatory Cytokines in Heart Failure: Double-Edged Swords. Heart Fail. Rev..

[15] Haudek S.B., Taffet G.E., Schneider M.D., Mann D.L. (2007). TNF Provokes Cardiomyocyte Apoptosis and Cardiac Remodeling through Activation of Multiple Cell Death Pathways. J. Clin. Investig..

[16] Chen F., Chang R., Trivedi M., Capetanaki Y., Cryns V.L. (2003). Caspase Proteolysis of Desmin Produces a Dominant-Negative Inhibitor of Intermediate Filaments and Promotes Apoptosis. J. Biol. Chem..

[17] Panagopoulou P., Davos C.H., Milner D.J., Varela E., Cameron J., Mann D.L., Capetanaki Y. (2008). Desmin Mediates TNF-Alpha-Induced Aggregate Formation and Intercalated Disk Reorganization in Heart Failure. J. Cell Biol..

[18] Caulín C., Salvesen G.S., Oshima R.G. (1997). Caspase Cleavage of Keratin 18 and Reorganization of Intermediate Filaments during Epithelial Cell Apoptosis. J. Cell Biol..

[19] Byun Y., Chen F., Chang R., Trivedi M., Green K.J., Cryns V.L. (2001). Caspase Cleavage of Vimentin Disrupts Intermediate Filaments and Promotes Apoptosis. Cell Death Differ..

[20] Zhang L., Kavanagh B.D., Thorburn A.M., Camidge D.R. (2010). Preclinical and Clinical Estimates of the Basal Apoptotic Rate of a Cancer Predict the Amount of Apoptosis Induced by Subsequent Proapoptotic Stimuli. Clin. Cancer Res..

[21] Linder S., Olofsson M.H., Herrmann R., Ulukaya E. (2010). Utilization of Cytokeratin-Based Biomarkers for Pharmacodynamic Studies. Expert Rev. Mol. Diagn..

[22] Elliott P., Andersson B., Arbustini E., Bilinska Z., Cecchi F., Charron P., Dubourg O., Kühl U., Maisch B., McKenna W.J. (2008). Classification of the Cardiomyopathies: A Position Statement from the European Society Of Cardiology Working Group on Myocardial and Pericardial Diseases. Eur. Heart J..

[23] Mitchell C., Rahko P.S., Blauwet L.A., Canaday B., Finstuen J.A., Foster M.C., Horton K., Ogunyankin K.O., Palma R.A., Velazquez E.J. (2019). Guidelines for Performing a Comprehensive Transthoracic Echocardiographic Examination in Adults: Recommendations from the American Society of Echocardiography. J. Am. Soc. Echocardiogr..

[24] Lang R.M., Badano L.P., Mor-Avi V., Afilalo J., Armstrong A., Ernande L., Flachskampf F.A., Foster E., Goldstein S.A., Kuznetsova T. (2015). Recommendations for Cardiac Chamber Quantification by Echocardiography in Adults: An Update from the American Society of Echocardiography and the European Association of Cardiovascular Imaging. J. Am. Soc. Echocardiogr..

[25] Robinson S., Ring L., Oxborough D., Harkness A., Bennett S., Rana B., Sutaria N., Lo Giudice F., Shun-Shin M., Paton M. (2024). The Assessment of Left Ventricular Diastolic Function: Guidance and Recommendations from the British Society of Echocardiography. Echo Res. Pract..

[26] Arbelo E., Protonotarios A., Gimeno J.R., Arbustini E., Barriales-Villa R., Basso C., Bezzina C.R., Biagini E., Blom N.A., de Boer R.A. (2023). 2023 ESC Guidelines for the Management of Cardiomyopathies. Eur. Heart J..

[27] O’Mahony C., Jichi F., Pavlou M., Monserrat L., Anastasakis A., Rapezzi C., Biagini E., Gimeno J.R., Limongelli G., McKenna W.J. (2014). A novel clinical risk prediction model for sudden cardiac death in hypertrophic cardiomyopathy (HCM risk-SCD). Eur. Heart J..

[28] Cui H., Schaff H.V., Lentz Carvalho J., Nishimura R.A., Geske J.B., Dearani J.A., Lahr B.D., Lee A.T., Bos J.M., Ackerman M.J. (2021). Myocardial Histopathology in Patients with Obstructive Hypertrophic Cardiomyopathy. J. Am. Coll. Cardiol..

[29] Polari L., Alam C.M., Nyström J.H., Heikkilä T., Tayyab M., Baghestani S., Toivola D.M. (2020). Keratin Intermediate Filaments in the Colon: Guardians of Epithelial Homeostasis. Int. J. Biochem. Cell Biol..

[30] Jerše M., Zidar N. (2011). Apoptosis in the Developing Human Heart Resembles Apoptosis in Epithelial Tissues. Cell Tissue Res..

[31] Senturk T., Aydinlar A., Yilmaz Y., Oral A.Y., Ozdabakoglu O., Ulukaya E. (2009). Serial Changes in Circulating M30 Antigen, a Biomarker of Apoptosis, in Patients with Acute Coronary Syndromes: Relationship with the Severity of Coronary Artery Disease. Coron. Artery Dis..

[32] Adlbrecht C., Hoetzenecker K., Posch M., Steiner S., Kopp C., Hacker S., Auer J., Horvath R., Moser B., Roth G. (2007). Elevated Levels of Interleukin-1β-Converting Enzyme and Caspase-Cleaved Cytokeratin-18 in Acute Myocardial Infarction. Eur. J. Clin. Investig..

[33] Mattey D.L., Dawes P.T., Nixon N.B., Goh L., Banks M.J., Kitas G.D. (2004). Increased Levels of Antibodies to Cytokeratin 18 in Patients with Rheumatoid Arthritis and Ischaemic Heart Disease. Ann. Rheum. Dis..

[34] Herzer K., Kneiseler G., Bechmann L.P., Post F., Schlattjan M., Sowa J.-P., Neumann T., Marggraf G., Erbel R., Gerken G. (2011). Onset of Heart Failure Determines the Hepatic Cell Death Pattern. Ann. Hepatol..

[35] Yang M.-C., Liu H.-K., Su Y.-T., Tsai C.-C., Wu J.-R. (2019). Serum Apoptotic Marker M30 Is Positively Correlated with Early Diastolic Dysfunction in Adolescent Obesity. PLoS ONE.

[36] Cambronero F., Marín F., Roldán V., Hernández-Romero D., Valdés M., Lip G.Y.H. (2009). Biomarkers of Pathophysiology in Hypertrophic Cardiomyopathy: Implications for Clinical Management and Prognosis. Eur. Heart J..

[37] Zen K., Irie H., Doue T., Takamiya M., Yamano T., Sawada T., Azuma A., Matsubara H. (2005). Analysis of Circulating Apoptosis Mediators and Proinflammatory Cytokines in Patients with Idiopathic Hypertrophic Cardiomyopathy: Comparison between Nonobstructive and Dilated-Phase Hypertrophic Cardiomyopathy. Int. Heart J..

[38] Sato Y., Taniguchi R., Nagai K., Makiyama T., Okada H., Yamada T., Matsumori A., Takatsu Y. (2003). Measurements of Cardiac Troponin T in Patients with Hypertrophic Cardiomyopathy. Heart.

[39] Dai L., Yan Y., Chen Q. (2024). Clinical Significance of Serum Ck18-M65 and M30 Levels in Patients with Chronic Hepatitis B Combined with Nonalcoholic Steatohepatitis and Liver Fibrosis. Medicine.

[40] Turk H.M., Aliyev A., Celik R.S., Seker M., Coban E., Demir T., Baydas T., Kocyigit A. (2020). Usefulness of Serum M30 and M65 Levels to Predict Response to Neoadjuvant Chemotherapy in Patients with Breast Cancer. Curr. Probl. Cancer.

[41] Koch A., Yagmur E., Linka J., Schumacher F., Bruensing J., Buendgens L., Herbers U., Koek G.H., Weiskirchen R., Trautwein C. (2018). High Circulating Caspase-Cleaved Keratin 18 Fragments (M30) Indicate Short-Term Mortality in Critically Ill Patients. Dis. Markers.

[42] Oweira H., Sadeghi M., Volker D., Mieth M., Zidan A., Khajeh E., Ghamarnejad O., Fonouni H., Weiss K.H., Schmidt J. (2018). Serum Caspase-Cleaved Cytokeratin (M30) Indicates Severity of Liver Dysfunction and Predicts Liver Outcome. Ann. Transplant..

[43] Xiong Y., Gao S., Luo G., Cheng G., Huang W., Jiang R., Wang Y., Cui T. (2017). Increased Circulating Autoantibodies Levels of IgG, IgA, IgM Against Cytokeratin 18 and Cytokeratin 19 in Chronic Obstructive Pulmonary Disease. Arch. Med. Res..

[44] Simopoulos C., Tsaroucha A.K., Asimakopoulos B., Giatromanolaki A., Gavriilidis P., Polychronidis A., Karayiannakis A. (2008). Total and Caspase-Cleaved Cytokeratin 18 in Chronic Cholecystitis: A Prospective Study. BMC Gastroenterol..

[45] Roth G.A., Krenn C., Brunner M., Moser B., Ploder M., Spittler A., Pelinka L., Sautner T., Wolner E., Boltz-Nitulescu G. (2004). Elevated Serum Levels of Epithelial Cell Apoptosis-Specific Cytokeratin 18 Neoepitope M30 in Critically Ill Patients. Shock.

[46] Högye M., Mándi Y., Csanády M., Sepp R., Buzás K. (2004). Comparison of Circulating Levels of Interleukin-6 and Tumor Necrosis Factor-Alpha in Hypertrophic Cardiomyopathy and in Idiopathic Dilated Cardiomyopathy. Am. J. Cardiol..

[47] Dirkx E., da Costa Martins P.A., De Windt L.J. (2013). Regulation of Fetal Gene Expression in Heart Failure. Biochim. Biophys. Acta.

