# Supplementary material for: Cytokeratin 18 as a Novel Biomarker in Patients with Hypertrophic Cardiomyopathy
Source: Cells. 2024 Aug 9;13(16):1328. doi: 10.3390/cells13161328 (PMC11352956; doi:10.3390/cells13161328)

Supplementary Data

Figure S1: A comparison of the expression of the CK18-based biomarkers in the HCM group vs. a normal group with 10 normal individuals**.**

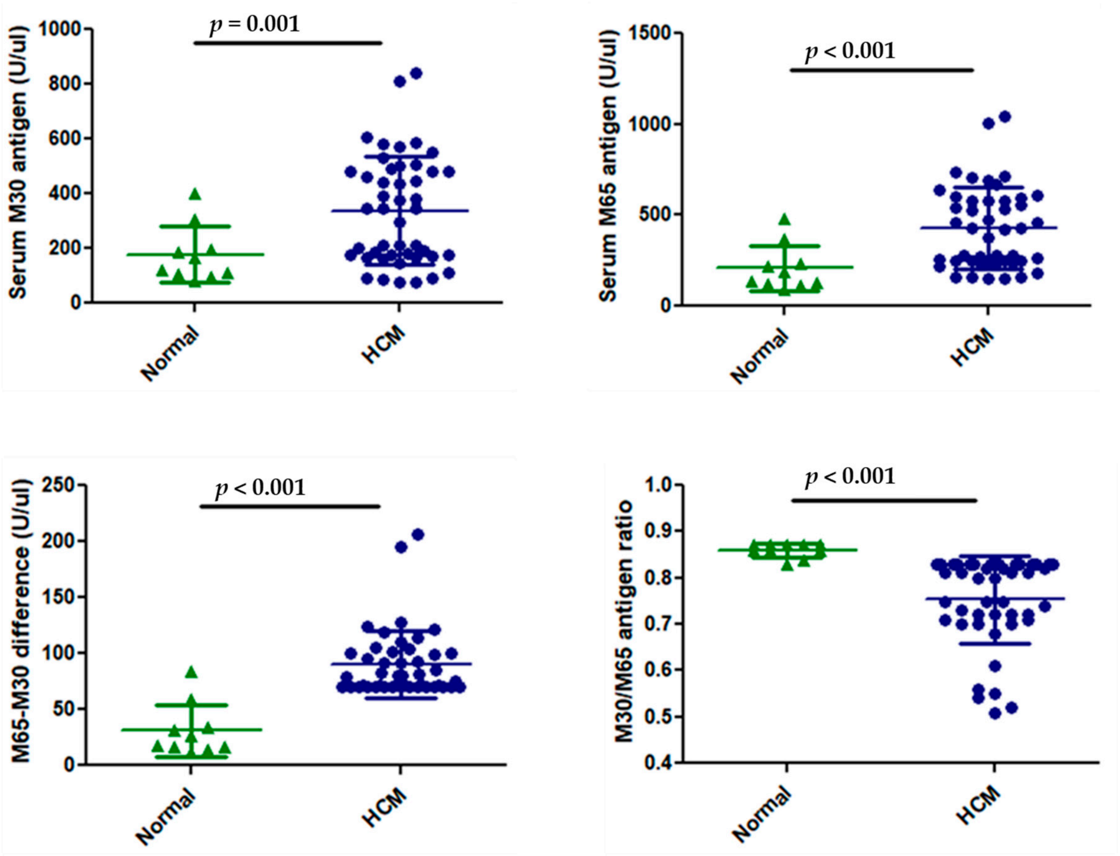

Table S1: Clinical characteristics of the post-mortem HCM or normal hearts and their associated quantitative data of CK18 immunostaining

	**HCM**	**Age (yrs)**	**Sex**	**HeartWeight (gr)**	**MWT (mm)**	**CAD**	**Cause of death**	**AREA**	**MEAN**	**MIN**	**MAX**	**% AREA**
**1**	No	29	Male	310	11	No	Headinjury	508,5	56,2	43	255	0,865
**2**	No	22	Female	251	10	No	HeadInjury	370,1	77,8	62,25	255	0,630
**3**	No	24	Male	313	11	No	HeadInjury	68,06	90,8	60	255	0,116
**4**	**Yes**	24	Male	390	16	No	Headinjury	941,1	68	44	255	1,602
**5**	**Yes**	20	Male	390	15	No	HeadInjury	736	76,8	59,75	255	1,253
**6**	**Yes**	45	Male	590	20	No	SCD	1404	48,6	34,33	255	2,389
**7**	**Yes**	20	Male	510	15	No	SCD	1056	81,8	56,5	255	1,797
**8**	**Yes**	28	Male	590	17	No	SCD	1056	69,8	49,71	255	1,796

Figure S2: The expression of CK18 in hepatocytes. Representative confocal images of hepatocytes, immunolabeled for cytokeratin 18 (red) and desmoplakin (green). Nuclei are stained with DAPI (blue). Scale bar:100 µm.

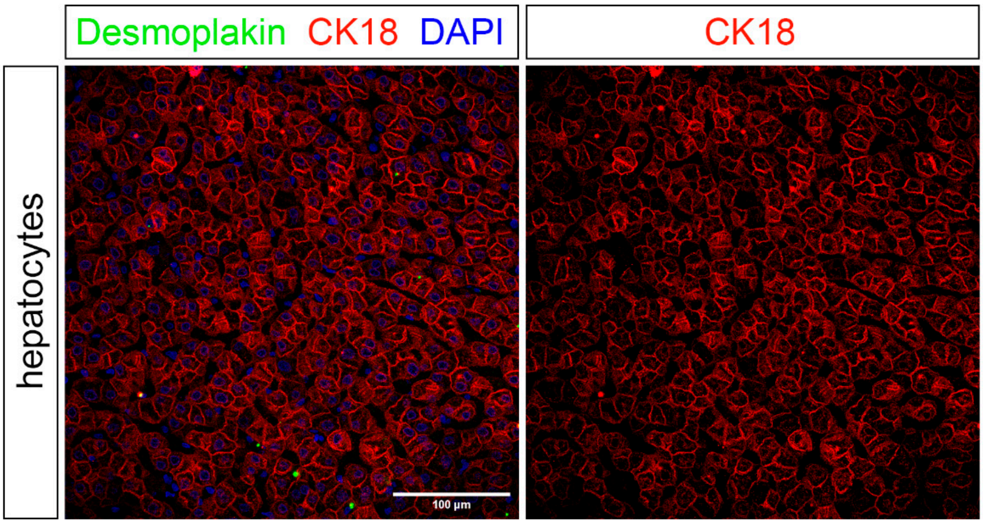

Supplement: Supplementary file 1 [file cells-13-01328-s001.zip › cells-3123563-supplementary.pdf]
